# A Multidimensional Strategy to Detect Polypharmacological Targets in the Absence of Structural and Sequence Homology

**DOI:** 10.1371/journal.pcbi.1000648

**Published:** 2010-01-22

**Authors:** Jacob D. Durrant, Rommie E. Amaro, Lei Xie, Michael D. Urbaniak, Michael A. J. Ferguson, Antti Haapalainen, Zhijun Chen, Anne Marie Di Guilmi, Frank Wunder, Philip E. Bourne, J. Andrew McCammon

**Affiliations:** 1 Biomedical Sciences Program, University of California San Diego, La Jolla, California, United States of America; 2 Department of Chemistry & Biochemistry, NSF Center for Theoretical Biological Physics, National Biomedical Computation Resource, University of California San Diego, La Jolla, California, United States of America; 3 San Diego Supercomputer Center and Department of Pharmacology, University of California San Diego, La Jolla, California, United States of America; 4 Division of Biological Chemistry and Drug Discovery, College of Life Sciences, University of Dundee, Dundee, United Kingdom; 5 Biocenter Oulu and Department of Biochemistry, University of Oulu, Oulu, Finland; 6 Institut de Biologie Structurale CEA; CNRS UMR 5075; Université Joseph Fourier, Partnership for Structural Biology, Grenoble, France; 7 Lead Discovery Wuppertal, Bayer Schering Pharma AG, Wuppertal, Germany; 8 Skaggs School of Pharmacy and Pharmaceutical Sciences, University of California San Diego, La Jolla, California, United States of America; 9 Howard Hughes Medical Institute, University of California San Diego, La Jolla, California, United States of America; European Molecular Biology Laboratory, Germany

## Abstract

Conventional drug design embraces the “one gene, one drug, one disease” philosophy. Polypharmacology, which focuses on multi-target drugs, has emerged as a new paradigm in drug discovery. The rational design of drugs that act *via* polypharmacological mechanisms can produce compounds that exhibit increased therapeutic potency and against which resistance is less likely to develop. Additionally, identifying multiple protein targets is also critical for side-effect prediction. One third of potential therapeutic compounds fail in clinical trials or are later removed from the market due to unacceptable side effects often caused by off-target binding. In the current work, we introduce a multidimensional strategy for the identification of secondary targets of known small-molecule inhibitors in the absence of global structural and sequence homology with the primary target protein. To demonstrate the utility of the strategy, we identify several targets of 4,5-dihydroxy-3-(1-naphthyldiazenyl)-2,7-naphthalenedisulfonic acid, a known micromolar inhibitor of *Trypanosoma brucei* RNA editing ligase 1. As it is capable of identifying potential secondary targets, the strategy described here may play a useful role in future efforts to reduce drug side effects and/or to increase polypharmacology.

## Author Summary

Proteins play a critical role in human disease; bacteria, viruses, and parasites have unique proteins that can interfere with human health, and dysfunctional human proteins can likewise lead to illness. In order to find cures, scientists often try to identify small molecules (drugs) that can inhibit disease-causing proteins. The goal is to identify a molecule that can fit snugly into the pockets and grooves, or “active sites,” on the protein's surface. Unfortunately, drugs that inhibit a single disease-causing protein are problematic. A single protein can evolve to evade drug action. Additionally, when only one protein is targeted, drug potency is often diminished. Single drugs that simultaneously target multiple disease-causing proteins are much more effective. On the other hand, if scientists are not careful, the drugs they design might inhibit essential human proteins in addition to inhibiting their intended targets, leading to unexpected side effects. In our current work, we have developed a computer-based procedure that can be used to identify proteins with similar active sites. Once unexpected protein targets have been identified, scientists can modify drugs under development in order to increase the simultaneous inhibition of multiple disease-causing proteins while avoiding potential side effects by decreasing the inhibition of useful human proteins.

## Introduction

Researchers have traditionally focused *in silico* efforts on designing inhibitors of specific protein targets, giving less attention to the computational identification of unpredicted secondary targets. This tendency is surprising given the frequency with which secondary receptors are responsible for both detrimental and beneficial pharmacological effects. The cost of developing a novel therapeutic ranges from $500 million to $2 billion dollars [1]. Millions of dollars are typically invested to advance a compound through clinical trials, but one third of these compounds fail or are later removed from the market due to unacceptable, medically harmful side effects [2] often caused by binding to off-target receptors. The detrimental effects caused by drug binding to unknown secondary targets can be financially and clinically devastating.

In other cases, compound binding to multiple therapeutic targets (polypharmacology) is beneficial. Conventional drug discovery embraces the “one gene, one drug, one disease” philosophy; however, drugs that target only one protein are susceptible to resistance, as a single amino-acid mutation in the target active site often substantially reduces compound binding affinity. Resistance to multi-target drugs, on the other hand, requires simultaneous mutations in multiple protein targets. Furthermore, drugs with polypharmacological mechanisms often have greater therapeutic potency. Some serotonergic drugs, for example, bind both 5-HT G-protein coupled receptors as well as the 5-HT_3A_ ion channel to achieve their therapeutic benefits, despite the fact that these two target proteins are not related by sequence or structure [3].

Identifying secondary targets in neglected tropical diseases, diseases for which drug development is neither profitable nor prevalent, allows researchers and doctors to retool approved drugs as novel treatments for the otherwise abandoned infections of the developing world. For instance, eflornithine, initially developed as an anti-cancer compound, was found to be a potent inhibitor of *Trypanosoma brucei* ornithine decarboxylase and is now a critical therapeutic in the fight against human African trypanosomiasis [4]. Examples like these motivate the need to develop new tools and algorithms to predict potential protein targets of candidate compounds.

A barrier to the development of these tools is the frequent absence of apparent evolutionary relationships among the multiple protein targets of a given compound, requiring that any potential method be capable of identifying target receptors independently of global sequence or structural homology. One approach is chemo-centric [5]; similar ligands are more likely to have similar properties and therefore often bind proteins with similar active sites. A number of studies have successfully identified secondary receptors by comparing their known small-molecule ligands [3], [6]–[8], leading to probabilistic models that can in some cases successfully predict polypharmacology. Despite these successes, however, chemo-centric approaches have their limitations. Chemically similar small molecules do not always inhibit proteins with similar active sites; indeed, even small changes in the chemical structures of some small-molecule inhibitors can drastically alter binding affinity [3] and the broad profile of binding to pharmacological targets.

A second approach is protein-centric. As the evolutionary relationships between secondary targets are not always apparent [3], receptor active-site geometries and pharmacophores must be compared directly, independently of global sequence or structural homology. Geometric constraints have been used extensively to identify binding sites and to assess binding-site similarity [9]–[16]. Of these methods, the sequence order independent profile-profile alignment (SOIPPA) algorithm is largely insensitive to both conformational changes in protein structure as well as the uncertainty inherent in homology models and low-resolution structures [17].

In the current work, we present a novel multidimensional strategy to identify the multiple protein receptors of a given compound that incorporates three levels of information: sequence-based homology clustering, the SOIPPA algorithm in conjunction with a geometric-potential metric [17]–[19], and *in silico* ligand docking. To demonstrate the utility of the strategy, we identify several human and pathogen secondary targets of compound **1** (NSC-45208), 4,5-dihydroxy-3-(1-naphthyldiazenyl)-2,7-naphthalenedisulfonic acid, a recently discovered micromolar inhibitor of *T. brucei* RNA editing ligase 1 (*Tb*REL1) [20]. *Tb*REL1 is a confirmed drug target in *T. brucei*, the causative agent of human African trypanosomiasis, a disease for which drug development has been largely neglected [21].

## Results

### Predicted Secondary Targets

**Figure 1:**
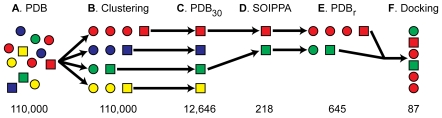
The strategy workflow. Circles and squares represent protein chains. Homologous chains share the same color. From each group of homologous chains, one is selected as representative and is shown as a square. (A) As the PDB has approximately 110,000 protein chains, identifying secondary targets directly is computationally intractable. (B) To reduce the number of chains, all chains are grouped by sequence homology into 12,646 clusters, and (C) a single representative chain is selected from each cluster. The set of all representative chains is called the PDB_30_. (D) SOIPPA is used to eliminate all protein chains in the PDB_30_ with active sites that are dissimilar to that of the primary target, *Tb*REL1. Only 218 chains remain. (E) A new set of 645 protein-chain structures called the PDB_r_ is created by taking the union of all those clusters whose representative PDB_30_ protein chains have active sites that are not dissimilar to that of *Tb*REL1. (F) Redundant chains are ignored; compound 1 is docked into the remaining 87 protein chains. Chains are ranked by their corresponding docking scores.

Sequence homology clustering was used to identify 12,646 protein-chain clusters from among the 110,000 protein chains present in the Protein Data Bank (PDB) as of late 2007 (Figure 1A, B). A representative protein chain was chosen from each cluster, creating a smaller set of chains called the PDB_30_ (Figure 1C). The SOIPPA algorithm in conjunction with a geometric-potential metric [17]–[19] revealed that the active sites of 12,428 of the representative PDB_30_ protein chains (98.3%) were dissimilar to that of *Tb*REL1 (*p*>0.05), the known target. After discarding these dissimilar protein chains, 218 representative PDB_30_ chains remained (Figure 1D). The remaining PDB_30_ proteins and the clusters they represented were merged into a single list containing 2,897 chains, a list enriched with possible secondary targets (Figure 1E). By considering only proteins from human or known human-pathogen species and eliminating PDBs with formatting errors, the number of chains was reduced from 2,897 to 645 (22.3%). This new list of protein chains was called the PDB_r_ (PDB_reduced_).

Compound **1**, a known inhibitor of the primary target, *Tb*REL1, was docked into each of the 645 potential secondary targets of the PDB_r_ with AutoDock 4.0 (Figure 1F). After docking, both protein chains of unknown function and chains belonging to proteins with duplicate names were deleted without regard for species. This pruning produced a list of 87 non-redundant predicted secondary targets. The docked pose of **1** into each of these 87 predicted secondary targets was analyzed to confirm binding to a pocket of known biochemical or pharmacological activity. In two instances, SITE data was included in the published PDB file, allowing us to identify one active-site and one off-site docking. In 25 instances, the docked ligand bound at the same location as a co-crystallized ligand, typically superimposed on top of it. Homology modeling revealed that an additional nine docked ligands bound at active sites of known biochemical or pharmacological activity, while 34 bound at alternate sites on the receptor. Though these alternate sites may be involved in allosteric regulation of protein function, we chose not to pursue them further. In all, the list of 87 protein chains contained 35 chains whose known active sites contained docked ligands, 35 chains whose alternate sites contained docked ligands, and 17 chains that could not be classified (Text S1).

### Experimental Validation of Predicted Secondary Targets

The predicted secondary targets that gave the best docking scores, *H. sapiens* mitochondrial 2-enoyl thioester reductase (*Hs*ETR1), *T. brucei* UDP-galactose 4′ epimerase (*Tb*GalE), *H. sapiens* phosphodiesterase 9A (*Hs*PDE9A2), and *Streptococcus pneumoniae* teichoic acid phosphorylcholine esterase (*Sp*Pce), were subsequently tested experimentally.

Compound **1** inhibited *Hs*ETR1 with a measured IC_50_ of approximately 33.5 µM and a Hill slope of 1.06. Neither the FATCAT algorithm nor CLUSTALW2 judged *Hs*ETR1 to be significantly homologous in sequence or structure to the primary target, *Tb*REL1 (*p* = 0.754; identity = 4%). In contrast, the SOIPPA algorithm judged the active sites of these two proteins to be significantly similar (*p*<1×10^−5^) (Table 1). *Hs*ETR1 aggregates were not detected, as measured by both spectrometry and centrifugation. A mixture of compound **1** and *Hs*ETR1 was run through a gel filtration column, thereby removing any unbound ligand. Spectroscopic analysis subsequently demonstrated that **1** was not covalently bound to *Hs*ETR1.

**Table 1:** Selected predicted secondary targets of compound 1 in humans.

	Protein	AD Score	SOIPPA *p*-value	Sequence Identity	FATCAT *p*-value
Metabolism	UDP-glucose 4-epimerase (1I3L:B)	−11.22		3%	0.613
	NAGK (2CH5:C)	−10.75	3.45×10^−2^	9%	0.351
	acetyl-CoA carboxylase 2 (2HJW:A)	−10.28	7.82×10^−3^	5%	0.423
	mitochondrial 2-enoyl thioester reductase (1ZSY:A)	−10.04	<1×10^−5^	4%	0.754
	tubby isoform A (1S31:A)	−9.17		6%	0.615
DNA synthesis, repair, replication	DNA ligase I (1X9N:A, residues 535–751)	−9.70	<1×10^−5^	5%	3.82×10^−3^
	3-methyl-adenine DNA glycosylase (1EWN:A)	−9.09	1.18×10^−2^	10%	0.800
	thymidylate synthase (1I00:A)	−8.50		3%	0.237
Amino acid synthesis	pyrroline-5-carboxylate reductase 1 (2GR9:B)	−10.49	4.28×10^−2^	1%	0.702
Blood clotting	fibrinogen (1FZE:B)	−9.53		4%	0.626
Vision	tubby related 1 (2FIM:B)	−10.21	2.60×10^−2^	3%	0.637
Nuclear transport	snurportin-1 (1XK5:A)	−10.10	<1×10^−5^	4%	6.07×10^−4^

Human secondary targets are involved in metabolism; polynucleotide synthesis, repair, and replication; amino acid synthesis; blood clotting; vision; and nuclear transport. “AD score” refers to the AutoDock-predicted energy of binding to 1; “SOIPPA *p*-value” refers to the similarity between the secondary-target and *Tb*REL1 active sites; “sequence identity” refers to the percent amino-acid identity with *Tb*REL1 as computed by the CLUSTALW2 algorithm; and “FATCAT *p*-value” refers to the structural similarity between the secondary target and *Tb*REL1. Protein sequences were extracted from PDB *seqres* headers.

Compound **1** inhibited *Tb*GalE with a measured IC_50_ of 0.7±0.2 µM and a Hill slope of 1.13+/−0.36. Again, the FATCAT algorithm did not judge the structure of *Tb*GalE to be significantly similar to that of the primary target, *Tb*REL1 (*p* = 0.627), and the CLUSTALW2 algorithm did not identify significant sequence homology (identity = 1%) (Table 2). *Tb*GalE inhibition was unaffected by the presence of detergent, and activity could be restored by dialysis of the protein, demonstrating that the *Tb*GalE inhibition was not due to aggregation of the compound or chemical modification of the protein.

**Table 2:** Selected predicted secondary targets of compound 1 in pathogens.

Protein	Species	AD Score	SOIPPA *p*-value	Sequence Identity	FATCAT *p*-value
probable ATP-dependent DNA ligase (2FAO:A)	*Pseudomonas aeruginosa*	−10.57	1.34×10^−2^	5%	0.440
UDP-galactose 4-epimerase (1GY8:D)	*T. brucei*	−10.29		1%	0.622
dTDP-D-glucose 4,6-dehydratase (1KET:B)	*Streptococcus suis*	−9.55		5%	0.502
dihydrofolate reductase-thymidylate (1J3I:C)	*Plasmodium falciparum*	−9.53	1.34×10^−2^	5%	0.428
DNA ligase, NAD-dependent (1TAE:B)	*E. faecalis v583*	−9.49		8%	3.56×10^−2^
dTDP-D-glucose 4,6-dehydratase (1G1A:C)	*S. enterica*	−9.24		1%	0.529
adenine phosphoribosyltransferase (1MZV:A)	*Leishmania tarentolae*	−8.61		11%	0.649
UTP-gluc-1-P uridylyltransferase 2 (2OEG:A)	*Leishmania major*	−8.56	7.82×10^−3^	8%	0.724
purine nucleoside phosphorylase (2B94:A)	*Plasmodium knowlesi*	−7.61		2%	0.650
DNA ligase (1ZAU:A)	*M. tuberculosis*	−6.75		4%	2.82×10^−2^

“AD score” refers to the AutoDock-predicted energy of binding to 1; “SOIPPA *p*-value” refers to the similarity between the secondary-target and *Tb*REL1 active sites; “sequence identity” refers to the percent amino-acid identity with *Tb*REL1 as computed by the CLUSTALW2 algorithm; and “FATCAT *p*-value” refers to the structural similarity between the secondary target and *Tb*REL1. Protein sequences were extracted from PDB *seqres* headers.

Two of the predicted secondary targets, *Hs*PDE9A2 and *Sp*Pce, were uninhibited by **1** at 200 µM and 10 mM, respectively. AutoDock predicted that **1** would bind *Hs*PDE9A2 and *Sp*Pce with −18.19 and −28.00 kcal/mol, respectively (Tables 1 and 2).

## Discussion

In this work, we attempt to further the study of polypharmacological and side-effect prediction by presenting a multidimensional strategy for identifying secondary targets of known enzyme inhibitors in the absence of global structure and sequence homology. To demonstrate the utility of the strategy, we identify secondary targets of **1**, a recently discovered inhibitor of *Tb*REL1 [20] from *T. brucei*, the causative agent of African sleeping sickness. *Tb*REL1 plays a critical role in the editing of trypanosomatid mitochondrial RNA transcripts and is required for the survival of both the *T. brucei* insect and bloodstream forms [22],[23]. Additionally, *Tb*REL1 is a particularly attractive drug target because there are no known close human homologs [20].

Compound **1** was chosen to illustrate how the current strategy can be applied early in the drug-discovery process. The compound inhibits a known drug target, satisfies Lipinski's rule of five, and is structurally similar to surinam, a drug currently approved for the treatment of human African trypanosomiasis. In this sense, **1** is drug like. Compound **1** has not yet been optimized to bind *Tb*REL1 in the nanomolar regime, however, and does contain some undesirable functional groups, and so is still very much under development. By incorporating the identification of secondary targets early in the drug-design process, we hope to eventually make modifications to compound **1** that will increase the binding affinity to the primary target while decreasing binding to undesirable secondary targets.

### Human Secondary Targets

Of the 35 predicted secondary targets of compound **1** identified, twelve were human proteins. Potential side effects of **1** can be predicted by considering the physiological role of these targets. For instance, a number of the predicted secondary human targets regulate metabolism, including the experimentally confirmed secondary target *Hs*ETR1. Neither FATCAT nor CLUSTALW2 judged *Hs*ETR1to be homologous to the primary target, *Tb*REL1 (Table 1). The current strategy, which is not dependent on sequence or global structural homology, was able to identify this secondary target where identification by homology would have failed. *Hs*ETR1 is thought to be essential for fatty acid synthesis (FAS) type II [24]. In the process of optimizing **1** to make it more druglike, modifications that reduce binding to human *Hs*ETR1 may diminish unforeseen side effects. Interestingly, AutoDock predicted that **1** partially occupies a co-factor (NADPH) binding site, suggesting that the compound may function as a competitive inhibitor for NADPH (Figure S1A).

*H. sapiens* UDP-galactose 4-epimerase (*Hs*GalE), a second protein involved in human metabolism, was also identified as a potential secondary target. Though *Hs*GalE shares little homology with the primary target, *Tb*REL1 (FATCAT *p*-value: 0.610; CLUSTALW2 identity: 2%), it is highly homologous with *Tb*GalE (FATPAT *p*-value: 0.00; CLUSTALW2 identity: 37%; Figure S2A), which we show to be a secondary target of **1** (IC_50_: 0.7±0.2 µM). Mutations in the *Hs*GalE gene cause type 3 galactosemia in humans. As toxic levels of galactose build up in patients' blood, vomiting, hepatomegaly, jaundice, renal failure, and cataracts typically follow [25]. Chronic administration of **1** is thus ill advised, though short-term treatment may be acceptable if patients avoid dairy and other sources of galactose.

In addition to metabolism, a number of the predicted human secondary targets are involved in polynucleotide synthesis, repair, and replication. *H. sapiens* DNA ligase I (*Hs*LigI), a protein that belongs to the same enzyme superfamily as *Tb*REL1, is one notable example. *Hs*LigI catalyzes the ultimate, essential step in DNA replication, repair, and recombination [26]. Studies have demonstrated that *Hs*LigI is defective in at least one representative lymphoid cell line of Bloom's syndrome origin [27], and a mouse model with a mutant *Hs*LigI allele exhibits an increased incidence of spontaneous cancer [28].

Though not tested explicitly, additional evidence does suggest that **1** binds *Hs*LigI. First, **1** is known to inhibit *H. sapiens* DNA ligase IIIβ (*Hs*LigIIIβ; IC_50_: 27.49±6.40 µM) [20], a *Hs*LigI homolog (Table 3). Second, the FATCAT algorithm [29] judged the structure of *Hs*LigI to be significantly similar to that of the primary target, *Tb*REL1, and the SOIPPA algorithm judged the active sites of these two proteins to be similar (Table 1). Finally, the AutoDock-predicted binding energy of **1** to *Hs*LigI was −9.70 kcal/mol (Table 1).

**Table 3:** Global sequence and structural homology between *Hs*LigI, residues 535 to 751, and three other selected DNA ligases.

	Identity Similarity	FATCAT *p* value
*Hs*LigIIIβ	31%	-
*E. faecalis* v583 NAD-dependent DNA ligase	4%	2.36×10^−7^
*M. tuberculosis* DNA ligase	4%	1.07×10^−6^

### Secondary Targets in Bacterial and Parasitic Pathogens

Of the 35 predicted secondary targets identified, 23 belonged to bacterial and parasitic species. Among these predicted pathogenic secondary targets, enzymatic assays demonstrated that **1** inhibits *Tb*GalE [30] (Table 2) with high nanomolar affinity. Interestingly, the FATCAT algorithm did not judge the structure of *Tb*GalE to be significantly similar to that of the primary target, *Tb*REL1 (Table 2), nor did CLUSTALW2 suggest sequence homology (identity = 1%). Thus, had we attempted to identify secondary targets by protein sequence or structural homology alone, *Tb*GalE would not have been detected. This result again illustrates the power of the current strategy.

Like *Tb*REL1, *Tb*GalE is essential for *T. brucei* survival [31]. Thus, **1** inhibits two essential *T. brucei* enzymes, an example of potential polypharmacology. In the process of optimizing **1** to make it more druglike, modifications that increase binding to both *Tb*REL1 and *Tb*GalE would likely improve drug efficacy and decrease the chances of resistance through mutation. Interestingly, AutoDock again predicted that **1** would bind partially in the *Tb*GalE NAD^+^ pocket, suggesting that the compound may be a competitive inhibitor for NAD^+^ rather than UDP-galactose (Figure S1B).

Though not tested explicitly, another predicted secondary target in pathogens, *Salmonella enterica* dTDP-D-glucose 4,6 dehydratase (*Se*RmlB), shares sequence and structural homology with *Tb*GalE and may also bind **1** (Table 2, Figure S2A). The FATCAT algorithm judged *Se*RmlB to be significantly structurally similar to *Tb*GalE (*p* value = 0.00), and CLUSTALW2 identified some sequence homology (identity = 21%). Additionally, AutoDock predicted that *Se*RmlB would bind **1** with relatively high affinity (Table 2).

*Se*RmlB is the second enzyme in the dTDP-L-rhamnose biosynthetic pathway; L-rhamnose is part of the LPS endotoxin (the O antigen) in many serotypes and serovars of Gram-negative bacteria. As L-rhamnose is common in the cell walls and envelopes of some pathogenic bacteria but absent in humans, *Se*RmlB is thought to be a potential drug target [32]. These findings support the hypothesis that compounds similar to **1** may have anti-bacterial properties.

Aside from inhibiting an essential protein in the bacterial dTDP-L-rhamnose biosynthetic pathway, the current strategy also identified two bacterial DNA ligases as potential secondary targets. *Enterococcus faecalis v583* NAD-dependent DNA ligase (*Ef* ligase) and *Mycobacterium tuberculosis* DNA ligase (*Mt* ligase) are structurally homologous to *Hs*LigI (Table 3, Figure S2B). Human DNA ligases require ATP as a co-factor, but bacterial ligases require NAD^+^ [33]. Because of this important biochemical difference, bacterial ligases are thought to be good drug targets.

### Method Limitations

A limitation of the current method is the prediction of false positives. Two of the predicted secondary targets, *Sp*Pce and *Hs*PDE9A2, were uninhibited by **1**. Closer inspection of the docking results revealed electrostatic energy components of −30.89 kcal/mol and −12.29 kcal/mol, respectively. In both cases, the partial charges of several active-site metal cations had been manually set to the corresponding formal charge. Subsequent analysis of the docked poses revealed that for both receptors, one of the sulfonate groups of **1** was juxtaposed against these highly charged metal cations (Figure S3). Clearly, a more careful treatment of the electrostatic interactions that accounts for electron polarization is warranted when docking into active sites that include metal ions.

An additional limitation of the current method is its dependence on sequence-homology clustering. Because of computational limitations, the number of potential secondary targets analyzed with SOIPPA had to be reduced; consequently, rather than analyzing all protein chains in the PDB, representative chains were selected from clusters of homologous proteins based on the supposition that sequence-homologous proteins would likely have similar active sites. This supposition, however, is hardly a universal truth. Through convergent evolution, two proteins with very different primary sequences may have evolved to bind similar ligands, and so may have similar active sites despite a lack of sequence homology.

Fortunately, recent advances in the SOIPPA algorithm now make sequence-homology clustering unnecessary. The version of SOIPPA used in the current study estimated the statistical significance of each active-site comparison using a non-parametric statistics method that required at least several hundred additional comparisons to derive a background distribution. Recently, an extreme-value distribution model has been developed to compute the statistical significance of SOIPPA scores [34]; this model improves the speed of the algorithm by at least two orders of multitude, so that each active-site comparison can be performed in mere seconds. Ligand binding-site similarity searches can now be performed on a genome-wide scale without the need for sequence-homology clustering [34],[35]. The new statistical model has been implemented in SMAP v2.0, available at http://funsite.sdsc.edu.

The manual verification of predicted compound binding to active sites of known biochemical or pharmacological activity also presented a limiting challenge. This step was very time consuming; had multiple compounds been tested, manual verification of all docked poses may have been impossible. To automate the process, active-site binding can be confirmed in many cases based on the proximity of the docked ligand to known catalytic residues annotated in the Catalytic Site Atlas [36] or to active-site residues specified in the site records of the PDB. Additionally, the SOIPPA implementation in SMAP v2.0, not available until recently, suggests an initial ligand binding pose for each predicted secondary target. Docked poses could be compared to this initial suggestion using an automated script and rejected in the absence of proximity.

### Accounting for Possible Promiscuous Binding

As experimental validation has confirmed that **1** inhibits multiple protein targets, the possibility of nonspecific, promiscuous binding must be eliminated. Previous work has demonstrated that compound **1** inhibition of several ATP-dependent proteins depends on the degree of homology with the primary target, *Tb*REL1, suggesting that binding occurs *via* a specific rather than promiscuous mechanism [20]. Additionally, the lack of *Sp*Pce or *Hs*PDE9A2 inhibition further suggests that indiscriminant inhibition is unlikely.

Promiscuous inhibition can occur when a compound forms colloidal aggregates that inhibit indiscriminately. In one recent study, 95% of the inhibitors identified in a high-throughput screen were subsequently found to inhibit *via* a nonspecific aggregation-based mechanism [37]. In theory, the chances of aggregation are reduced in the case of **1** because of its negative charge; individual molecules should repel each other, preventing aggregation. To confirm this theory, Amaro et. al assayed **1** against *Tb*REL1 in the presence of a non-ionic detergent as well as an additional protein (BSA) known to prevent aggregation. The presence of the detergent and the separate test with BSA did not significantly influence *Tb*REL1 inhibition, demonstrating that **1** does not aggregate [20].

The current work likewise suggests no aggregation. The presence of detergent had no effect on *Tb*GalE inhibition. Additionally, no *Hs*ETR1 aggregation was observed, as measured by both spectrometry and centrifugation (see the Text S1). The Hill slopes corresponding to the inhibition of *Hs*ETR1 and *Tb*GalE were 1.06 and 1.13, respectively. As these slopes are approximately equal to one, the inhibition of these two proteins likely occurs *via* ligand binding to a single site, as predicted. One recent study suggested that aggregation-based inhibition typically produces Hill slopes that are much steeper, with average values of about 2.2 [37].

Promiscuous inhibition can also occur when compounds chemically modify the proteins they inhibit. Several experiments were performed in order to rule out chemical modification. To test for chemical modification of *Tb*GalE, *Tb*GalE was incubated with **1**, and subsequent dialysis was used to remove any unbound ligand. *Tb*GalE activity was unaffected following dialysis, as compared to treatment with DMSO alone. Had **1** been covalently linked to *Tb*GalE, the compound would not have been washed away, and the enzyme would have shown little activity.

To test for chemical modification of *Hs*ETR1, a mixture of compound **1** and *Hs*ETR1 was run through a gel filtration column, which likewise removed any unbound ligand. The absorption spectrum of the fraction containing *Hs*ETR1 was subsequently analyzed and did not demonstrate the peaks characteristic of **1** at 530 nm and 320 nm, likewise demonstrating that **1** was not covalently bound to *Hs*ETR1.

### A Cursory Chemo-Centric Analysis

A number of the predicted secondary targets identified belong to the same or similar biochemical pathways (i.e. metabolic pathways; DNA synthesis, repair, and replication pathways; and DNA ligase pathways) (Table 1). This result is encouraging, as proteins of the same pathway often act on similar substrates and so have similar active sites. A cursory chemo-centric look at the native substrates of the identified secondary targets in many instances corroborates our findings. For instance, the human proteins *Hs*LigI, 3-methyl-adenine DNA glycosylase, and thymidylate synthase, all involved in DNA synthesis, repair, and replication, were identified as potential target receptors. *Hs*LigI is a *Tb*REL1 homolog (Table 1) that ligates DNA in a way analogous to *Tb*REL1 dsRNA ligation. In contrast, 3-methyl-adenine DNA glycosylase is not a *Tb*REL1 homolog (Table 1), but an examination of its structure nevertheless reveals that it also binds DNA. One of the nucleotides of the bound DNA, an alkylated base generated endogenously by lipid peroxidation, protrudes into a deep binding pocket in a way analogous to *Tb*REL1-ATP binding [38]. Thymidylate synthase, another predicted secondary target involved in nucleotide synthesis, does not bind double-stranded nucleic acid, but rather binds deoxyuridine monophosphate, a compound with a nucleotide-ribose-phosphate substructure like that of ATP, a known substrate of the primary target, *Tb*REL1.

Notably, two experimentally validated secondary targets contain NAD^+^ or NADPH co-factors, and in both cases **1** is predicted to bind at least partially in one of the co-factor binding pockets. Similar to ATP, NAD^+^ and NADPH both contain adenine-ribose-biphosphate substructures. Of the 23 proteins listed in Tables 1 and 2, eight contain NAD^+^-like co-factors. We expect that there are even more true positives among the predicted secondary targets; to this end, we provide the entire list of predicted targets in Text S1.

Traditionally, researchers have devoted their computational efforts to designing inhibitors of specific protein targets while paying less attention to the *in silico* prediction of secondary targets. Because adverse side effects are often discovered late in the drug-development process, often after the investment of many millions of dollars, we recommend using the current strategy to help bad drugs “fail early,” or, better yet, to guide the drug-discovery process towards more selective inhibitors. Additionally, the current methodology can help medicinal chemists overcome the conventional “one gene, one drug, one disease” paradigm. The rational design of drugs that act *via* polypharmacological mechanisms can produce compounds that exhibit increased therapeutic potency and against which resistance is less likely to develop.

## Materials and Methods

### Compound Purity

Compound **1** was provided by the NCI/DTP Open Chemical Repository (http://dtp.cancer.gov). Compound identity was confirmed by high-resolution mass spectrometry, and no impurities were detected by ^1^H-NMR.

### Computational Methodology

The computational strategy presented here utilizes three distinct components in order to identify secondary pharmacological targets.

### 1. Homology Clustering: Creating a Non-Redundant PDB Representation

Identifying potential secondary targets from among the 110,000 protein chains deposited in the RCSB PDB [39] as of late 2007 was judged computationally intractable. In order to reduce the number of protein chains, redundancies in the RCSB PDB were eliminated by clustering all protein chains by sequence using the NCBI *blastclust* program, with a sequence identity threshold of 30% and an overlap threshold of 0.9 (Figure 1A, B). A representative protein chain was then chosen at random from each cluster, thus creating a smaller set of chains called the PDB_30_ (Figure 1C).

### 2. SOIPPA: Identifying Active Sites Similar to that of the Primary Target

In order to eliminate those members of the PDB_30_ whose active-site alpha-carbon configurations were different enough from that of the primary target, *Tb*REL1, so as to likely preclude NSC4520 binding, we used the SOIPPA algorithm in conjunction with a geometric potential [17]–[19], which computes general binding-site similarity based on shape (alpha-carbon tessellation), physical properties, and the evolutionary profiles of the active-site residues, without regard for specific side-chain positions or global sequence or structure (Figure 1D). Using SOIPPA and the geometric potential, we eliminated all protein chains in the PDB_30_ with active sites that were dissimilar to that of the primary target, *Tb*REL1 (*p*-value>0.05) (Figure 1D). The *p*-value was calculated from a non-parametric density function generated from 980 PDB chains with unique SCOP folds [18]. To derive the background distribution, a Gaussian function was placed at each observation. The mean of the Gaussian of the observed binding-site similarity score and its variance were fixed. The final probability density function was the sum of all these Gaussian functions. The optimal bandwidth was estimated from the data by using a least square cross-validation approach [40].

Each representative protein chain corresponded to a PDB cluster containing multiple homologous chains. A new set of protein-chain structures called the PDB_r_ (PDB_reduced_) was created by taking the union of all those clusters whose representative PDB_30_ protein chains had active sites that were not dissimilar to that of *Tb*REL1 (*p*-value<0.05) (Figure 1E). By considering only proteins from human or known human-pathogen species, the number of chains was significantly reduced. An additional protein, 1GJJ, was eliminated because of apparent PDB formatting errors. 1S31 was retained despite having a malformed GLU residue (561) to which Gasteiger partial charges could not be assigned.

### 3. *In silico* Docking

AutoDock 4.0 [41] was used to dock **1** into the protein chains of the PDB_r_ (Figure 1F). In previous work, AutoDock was validated against *Tb*REL1 [20]. To define the distinct docking grid associated with each protein chain, SOIPPA was used to identify the most probable active site, and the grid box was set to the smallest possible X-Y-Z cube encompassing all the atoms of the SOIPPA-reported alignable alpha carbons. Additional details regarding receptor and ligand preparation and grid and docking parameters can be found in Text S1.

All dockings were sorted by the predicted binding energy of their most-populated AutoDock clusters. After eliminating protein chains of unknown function from the list, the data was grouped according to species and protein, revealing significant redundancy in the PDB_r_. We selected the ligand-protein pair from each group with the best AutoDock score, producing a list of non-redundant ligand-protein pairs, one corresponding to each protein/species group (Text S1).

For each of these ligand-protein pairs, we used one of several methods to determine if AutoDock placed **1** in an active site of known biochemical or pharmacological activity. First, SITE data included in the published PDB file identified several active sites. Second, co-crystallized ligands bound in native active sites were examined. Finally, homology modeling was used to determine the locations of active sites for the remaining protein chains, when possible. A protein chain was considered to be a “hit” if **1** had a high predicted energy of binding and if **1** was predicted to bind in an identified active site of known biochemical or pharmacological activity. A description of the assays used to experimentally validate several of the predicted secondary targets is included in Text S1.

## Supporting Information

Text S1 Text S1 contains details about the computational methods employed and the assays used to confirm theoretical results. It also contains expanded versions of Tables 1 and 2.
(0.23 MB DOC)

Figure S1 Possible binding of compound 1 in NAD+ and NADPH pockets. In the case of two experimentally validated secondary targets, AutoDock predicted that 1 would bind in a NAD+ or NADPH pocket, suggesting that 1 may be a competitive inhibitor for these co-factors. (a) HsETR1. The crystal structure (PDB: 1ZSY) contained no NAD+ co-factor, so a related structure (PDB: 1GUF) with co-crystallized NDP (NADPH dihydro-nicotinamide-adenine-dinucleotide phosphate) was aligned to the 1ZSY structure using MultiSeq. The aligned NDP is shown in red. The predicted binding pose of 1 is shown in green. (b) TbGalE. The co-crystallized NAD+ co-factor is shown in red. The predicted binding pose of 1 is shown in green.
(3.98 MB TIF)

Figure S2 Unvalidated but likely secondary targets of compound 1. (a) The current strategy correctly identified TbGalE as a secondary target of 1. Additionally, HsGalE and SeRmlB, both TbGalE homologs, are also predicted to be off-target receptors. SeRmlB and TbGalE were aligned to HsGalE using MultiSeq to demonstrate structural similarity. Blue: TbGalE; Red: SeRmlB; Green: HsGalE. (b) The current strategy identified a number of DNA ligases as predicted secondary targets of 1. Three of these DNA ligases are homologous with HsLigIIIβ, an experimentally validated secondary target. The structures of the three predicted secondary targets were aligned using MultiSeq to demonstrate structural similarity. Portions of some ligands were removed to simplify visualization. The active site is shown with selected protein residues to demonstrate active-site similarity. Blue: HsLigI; Red: Ef ligase; Green: Mt ligase.
(3.65 MB TIF)

Figure S3 False-positive predictions. Compound 1 is shown in licorice, docked into each protein crystal structure. Magnesium and zinc are shown in green and grey, respectively. In both cases, one of the sulfonate groups of 1 is juxtaposed against multiple metal cations, leading to an exaggerated estimate of the electrostatic energy. (a) SpPce was predicted to bind 1 with −28.00 kcal/mol. (b) HsPDE9A2 was predicted to bind 1 with −18.19 kcal/mol.
(2.66 MB TIF)

## References

[1] Adams CP, Brantner VV (2006). Estimating the cost of new drug development: is it really 802 million dollars?. Health Aff (Millwood).

[2] Kennedy T (1997). Managing the drug discovery/development interface.. Drug discovery today.

[3] Keiser MJ, Roth BL, Armbruster BN, Ernsberger P, Irwin JJ (2007). Relating protein pharmacology by ligand chemistry.. Nat Biotechnol.

[4] Croft SL (2005). Public-private partnership: from there to here.. Trans R Soc Trop Med Hyg.

[5] Schreiber SL (2005). Small molecules: the missing link in the central dogma.. Nat Chem Biol.

[6] Paolini GV, Shapland RH, van Hoorn WP, Mason JS, Hopkins AL (2006). Global mapping of pharmacological space.. Nat Biotechnol.

[7] Vieth M, Higgs RE, Robertson DH, Shapiro M, Gragg EA (2004). Kinomics-structural biology and chemogenomics of kinase inhibitors and targets.. Biochim Biophys Acta.

[8] Izrailev S, Farnum MA (2004). Enzyme classification by ligand binding.. Proteins.

[9] Coleman RG, Sharp KA (2006). Travel depth, a new shape descriptor for macromolecules: application to ligand binding.. J Mol Biol.

[10] Nayal M, Honig B (2006). On the nature of cavities on protein surfaces: application to the identification of drug-binding sites.. Proteins.

[11] Coleman RG, Burr MA, Souvaine DL, Cheng AC (2005). An intuitive approach to measuring protein surface curvature.. Proteins.

[12] Agarwal PK, Edelsbrunner H, Harer J, Wang Y (2004). Extreme elevation on a 2-manifold... Symp Comp Geo.

[13] Hendrix DK, Kuntz ID (1998). Surface solid angle-based site points for molecular docking.. Pac Symp Biocomput.

[14] Liang J, Edelsbrunner H, Woodward C (1998). Anatomy of protein pockets and cavities: measurement of binding site geometry and implications for ligand design.. Protein Sci.

[15] Norel R, Wolfson HJ, Nussinov R (1999). Small molecule recognition: solid angles surface representation and molecular shape complementarity.. Comb Chem High Throughput Screen.

[16] Watson JD, Laskowski RA, Thornton JM (2005). Predicting protein function from sequence and structural data.. Curr Opin Struct Biol.

[17] Xie L, Bourne P (2007). A robust and efficient algorithm for the shape description of protein structures and its application in predicting ligand binding sites.. BMC Bioinformatics.

[18] Xie L, Bourne PE (2008). Detecting evolutionary relationships across existing fold space, using sequence order-independent profile-profile alignments.. Proc Natl Acad Sci U S A.

[19] Xie L, Wang J, Bourne PE (2007). In silico elucidation of the molecular mechanism defining the adverse effect of selective estrogen receptor modulators.. PLoS Comput Biol.

[20] Amaro RE, Schnaufer A, Interthal H, Hol W, Stuart KD (2008). Discovery of drug-like inhibitors of an essential RNA-editing ligase in Trypanosoma brucei.. Proceedings of the National Academy of Sciences.

[21] Remme JH, Blas E, Chitsulo L, Desjeux PM, Engers HD (2002). Strategic emphases for tropical diseases research: a TDR perspective.. Trends in parasitology.

[22] Schnaufer A, Panigrahi AK, Panicucci B, Igo RP, Salavati R (2001). An RNA Ligase Essential for RNA Editing and Survival of the Bloodstream Form of Trypanosoma brucei.. Science.

[23] Rusche LN, Huang CE, Piller KJ, Hemann M, Wirtz E (2001). The two RNA ligases of the Trypanosoma brucei RNA editing complex: cloning the essential band IV gene and identifying the band V gene.. Molecular and cellular biology.

[24] Miinalainen IJ, Chen ZJ, Torkko JM, Pirila PL, Sormunen RT (2003). Characterization of 2-enoyl thioester reductase from mammals. An ortholog of YBR026p/MRF1'p of the yeast mitochondrial fatty acid synthesis type II.. J Biol Chem.

[25] Rake J, Visser G, Smit G (2006). Disorders of Carbohydrate and Glycogen Metabolism.

[26] Pascal JM, O'Brien PJ, Tomkinson AE, Ellenberger T (2004). Human DNA ligase I completely encircles and partially unwinds nicked DNA.. Nature.

[27] Willis AE, Lindahl T (1987). DNA ligase I deficiency in Bloom's syndrome.. Nature.

[28] Harrison C, Ketchen AM, Redhead NJ, O'Sullivan MJ, Melton DW (2002). Replication failure, genome instability, and increased cancer susceptibility in mice with a point mutation in the DNA ligase I gene.. Cancer Res.

[29] Ye Y, Godzik A (2003). Flexible structure alignment by chaining aligned fragment pairs allowing twists.. Bioinformatics (Oxford, England).

[30] Shaw MP, Bond CS, Roper JR, Gourley DG, Ferguson MA (2003). High-resolution crystal structure of Trypanosoma brucei UDP-galactose 4′-epimerase: a potential target for structure-based development of novel trypanocides.. Mol Biochem Parasitol.

[31] Roper JR, Guther ML, Milne KG, Ferguson MA (2002). Galactose metabolism is essential for the African sleeping sickness parasite Trypanosoma brucei.. Proceedings of the National Academy of Sciences of the United States of America.

[32] Allard ST, Giraud MF, Whitfield C, Graninger M, Messner P (2001). The crystal structure of dTDP-D-Glucose 4,6-dehydratase (RmlB) from Salmonella enterica serovar Typhimurium, the second enzyme in the dTDP-l-rhamnose pathway.. J Mol Biol.

[33] Gajiwala KS, Pinko C (2004). Structural rearrangement accompanying NAD+ synthesis within a bacterial DNA ligase crystal.. Structure.

[34] Xie L, Bourne PE (2009). A unified statistical model to support local sequence order independent similarity searching for ligand-binding sites and its application to genome-based drug discovery.. Bioinformatics.

[35] Xie L, Li J, Bourne PE (2009). Drug discovery using chemical systems biology: identification of the protein-ligand binding network to explain the side effects of CETP inhibitors.. PLoS Comput Biol.

[36] Porter CT, Bartlett GJ, Thornton JM (2004). The Catalytic Site Atlas: a resource of catalytic sites and residues identified in enzymes using structural data.. Nucleic Acids Res.

[37] Feng BY, Simeonov A, Jadhav A, Babaoglu K, Inglese J (2007). A high-throughput screen for aggregation-based inhibition in a large compound library.. J Med Chem.

[38] Lau AY, Wyatt MD, Glassner BJ, Samson LD, Ellenberger T (2000). Molecular basis for discriminating between normal and damaged bases by the human alkyladenine glycosylase, AAG.. Proc Natl Acad Sci U S A.

[39] Berman HM, Westbrook J, Feng Z, Gilliland G, Bhat TN (2000). The Protein Data Bank.. Nucleic Acids Research.

[40] Silverman BW (1986). Density Estimation for Statistics and Data Analysis.

[41] Morris GM, Goodsell DS, Halliday RS, Huey R, Hart WE (1998). Automated docking using a Lamarckian genetic algorithm and an empirical binding free energy function.. Journal of Computational Chemistry.

