# Supplementary material for: A Multidimensional Strategy to Detect Polypharmacological Targets in the Absence of Structural and Sequence Homology
Source: PLoS Comput Biol. 2010 Jan 22;6(1):e1000648. doi: 10.1371/journal.pcbi.1000648 (PMC2799658; doi:10.1371/journal.pcbi.1000648)
Supplement: Text S1 — contains details about the computational methods employed and the assays used to confirm theoretical results. It also contains expanded versions of Tables 1 and 2. (0.23 MB DOC) [file pcbi.1000648.s001.doc]

**Supporting Information**

**Discussion**

*Tool Selection.* Though many methods of comparing active-site geometries have been proposed [1-8], SOIPPA was chosen because it is particularly robust. As it considers only the geometry of active-site alpha carbons, SOIPPA is largely insensitive to both conformational changes in protein structure as well as the uncertainties inherent in homology models and low-resolution structures [9]. SOIPPA finds the most similar local surface patch between two protein structures in the spirit of local sequence alignment, though the algorithm is independent of sequence order. This feature makes SOIPPA appropriate for practical problems; typically the boundary of a ligand binding site is not clearly defined and depends on the bound ligand. Additionally, SOIPPA does not require an all-atom representation of the protein structure, thus making it computationally efficient and scalable.

The freely distributed program AutoDock 4.0 [10] was used for docking because it employs a well tested, physics-based scoring function in conjunction with a Lamarkian genetic algorithm to improve accuracy [10,11]. Additionally, AutoDock has been used successfully in the past to identify *Tb*REL1 inhibitors [12]. The AutoDock scoring function has a standard error of 2.177 kcal mol^-1^ and is much faster, though less accurate, than comprehensive physics-based approaches like thermodynamic integration [10,13], single-step perturbation [14], and free energy of perturbation [15]. A recent validation study [16] showed that AutoDock performs well compared to other docking programs such as DOCK [17], FleX [18], and GOLD [19].

**Methods**

**Biochemical Assays**

*T. brucei UDP-Galactose 4' Epimerase (TbGalE) Assays.* Recombinant *T. brucei* UDP-Galactose 4'-epimerase (*Tb*GalE) containing an N-terminal hexahistidine tag was expressed in E. coli and purified as described previously [20]. The inhibition of *Tb*GalE was measured using high pH anion exchange chromatography (HPAEC) to follow the conversion of UDP-Gal to UDP-Glc by *Tb*GalE [21]. The reaction mixture (1 mM Tris pH 7.6, 100 mM UDP-Gal, 100 mM b-NAD+, 5 ug/mL *Tb*GalE, 1% dimethyl sulphoxide) was incubated at 37^o^C for 30 min with or without inhibitor, quenched with ten-fold excess of 1 mM NaOH, and then subjected to HPAEC chromatography on a CarboPac PA-1 column (Dionex) using conditions adapted from Tomiya et al. [22] The eluant was monitored at 260nm, and peaks assigned by comparison to commercial standards. The IC_50_ value was calculated using a four-parameter fit of eight-point potency curves derived from three independent experiments. When testing for the effect of detergent, 0.06% n-octylglucopyranoside was included in the reaction, and the quenched reaction mixture was passed through a C8 cartridge (Isolute) to remove detergent prior to analysis.

*S. pneumoniae Teichoic Acid Phosphorylcholine Esterase (SpPce) Assays.* The cloning, expression, and purification of choline binding protein E (CBPE) has been described previously [23]. The CBPE protein contains the catalytic phosphorylcholine esterase (*Sp*Pce) domain associated with the choline-binding domains. The Pce activity was measured using p-nitrophenyl-phosphorylcholine (pNP-PC, N5879, Sigma) as the substrate, at 37°C in 50 mM potassium phosphate buffer pH 8.0 in a total volume of 100 µl. The activity was measured by following the increase in absorbance at 405 nm (Fluostar Optima, BMG) due to the production of pNP, whose quantification as the reaction product of Pce activity had been previously calibrated with a standard curve. In the inhibition experiments, the enzyme and substrate concentrations were 0.5 µM and 8 µM, respectively, and the concentration of the **1** compound was varied from 0.2 mM to 10 mM.

*H. sapiens Mitochondrial 2-enoyl Thioester Reductase (HsETR1) Assays.* Human *Hs*ETR1 was cloned, expressed and purified as described previously [24,25]. To test the inhibitory effect of **1**, 0.5 µg of human recombinant *Hs*ETR1 was pre-incubated in the presence of **1** for five minutes with 125 µM NADPH and 0.1 µg/µl of bovine serum albumin in 50 mM KPi, pH 7.6 (at 22 ºC). The assay was started by adding 60 µM of (2E)-octenoyl-CoA (C8) substrate (Torkko & Koivuranta et al., Mol Cell Biol, 2001). C8 substrate was used because *Hs*ETR1 has shown the highest catalytic efficiency toward C8 and C12 [25]. The concentration of **1** was varied between 0 and 400 µM.

To test for aggregation of **1**, the absorbance of a mixture containing *Hs*ETR1, compound **1**, NADPH, and the substrate was monitored at 750 nm, a wavelength that none of the components absorbs. As there was no change in the absorbance at this wavelength, we conclude that there are no micro-aggregates/particles greater than roughly 375 nm in size. Because a wavelength of 340 nm was used to obtain IC_50_ values, we further conclude that there are no micro-aggregates/particles greater than roughly 170 nm in size. Visual inspection confirmed the absence of macro-aggregates.

As a second test for aggregation, two reaction mixtures were prepared, one containing *Hs*ETR1 and one containing *Hs*ETR1 + **1**. After 10 minutes incubation at room temperature, both tubes were centrifuged to separate any aggregates/particles from the supernatant. The concentration of soluble protein in the supernatant was then measured, and was found to be identical in both cases, again suggesting that **1** does not aggregate *Hs*ETR1.

*H. sapiens Phosphodiesterase 9A (HsPDE9A2) Assays.* Human PDE9A2 (accession no.: NM_002606) was recombinantly expressed in Sf9 insect cells using the pFASTBAC baculovirus expression system (Invitrogen Life Technologies, Carlsbad, CA, USA). Cells were harvested and resuspended in lysis buffer (20 ml/1l culture; 50 mM Tris/HCl pH 7.4, 50 mM NaCl, 1 mM MgCl_2_, 1.5 mM EDTA, 10% Glycerin). The cells were disrupted by sonication at 4°C and cell debris were removed by centrifugation at 15,000 x g at 4°C for 30 minutes. The supernatant was stored at -80°C.

The commercially available [^3^H]cAMP and [^3^H]cGMP Scintillation Proximity Assay (GE Healthcare, Little Chalfont, Buckinghamshire, UK) system was used for enzyme inhibition studies. For the determination of the in vitro effect of test substances on the PDE9A reaction, 2 µl of the respective test compound in dimethyl sulfoxide (serial dilutions) were placed in wells of microtiter plates (Isoplate; PerkinElmer Wallac, Weiterstadt, Germany). Fifty milliliters of a dilution of PDE9A cell extract in buffer A (50 mM Tris-HCl, pH 7.5, 8.3 mM MgCl2, 1.7 mM EDTA, and 0.2% BSA) was added. The dilution of the PDE9A cell extract was chosen such that the reaction kinetics were linear, and less than 70% of the substrate was consumed. The reaction was started by the addition of 50 µl (0.025 µCi) of 1:2000 in buffer A without BSA-diluted substrate, [8-^3^H]guanosine 3',5'-cyclic phosphate (1 µCi/µl; GE Healthcare). After incubation at room temperature for 60 min, the reaction was stopped by the addition of 25 µl of a suspension containing 18 mg/ml yttrium scintillation proximity beads (GE Healthcare) in water. 25 µl of a PDE9 inhibitor dissolved in buffer A without BSA was added before the addition of beads (BAY 73-6691, 5 µM final concentration). The microtiter plates were measured in a Microbeta scintillation counter (PerkinElmer Wallac). IC_50_ values were determined from sigmoidal curves by plotting the percentage of PDE activity versus log compound concentration. IC_50_ is defined as the concentration of the inhibitor required to reduce the cyclic nucleotide hydrolyzing activity of tested PDEs by 50%.

**Measuring Sequence and Structural Homology**

In order to assess sequence homology, the computer program CLUSTALW2 [26] was used to align each protein amino-acid sequence to that of *Tb*REL1. Protein sequences were extracted from the RCSD PDB [27], except for the sequence of *H. sapiens* DNA Ligase IIIβ, which was obtained from the *UniProt* database [28]. Percent identity scores are reported in Tables 1, 2, and 3.

To assess structural homology, the FATCAT algorithm [29] was used to align each protein structure with that of *Tb*REL1. FATCAT is an algorithm for flexible structure alignment that minimizes the number of rigid-body movements (twists) and pivot points (hinges) introduced in the reference protein. In all cases, the flexible alignment model was used and the measure of similarity, *p*, was reported (Tables 1, 2, and 3), where *p* < 0.05 indicates significant structural similarity. For the purpose of visualization (Fig. S2), proteins were aligned using *MultiSeq* [30] and visualized in VMD [31].

**Docking Parameters**

To prepare each receptor PDB file for docking, all co-crystallized waters, non-metallic ions, and ligands were removed, and additional side-chain rotamers, if reported, were eliminated. All selenomethionine (MSE) residues were replaced with methionine (MET) residues by changing the selenium atom to a sulfur atom. The PDB file was subsequently processed with the AutoDock 4.0 receptor preparation script, which computes Gasteiger charges and adds non-polar hydrogen atoms. Partial charges for metal ions were set to the formal charge manually. Grid spacing was set to 0.375 Å, and for each protein receptor eight affinity maps were calculated: A (aromatic carbon), C, HD (donor 1 H-bond hydrogen), N, NA (hydrogen-bond-accepting N), OA (hydrogen-bond-accepting O), S, and e (electrostatic).

The PDB file of **1** was retrieved from the NCI website and processed with AutoDockTools version 1.4.5 to add missing hydrogen atoms, compute Gasteiger partial charges for each atom, and merge non-polar hydrogen atoms. During docking all torsions were assigned with the AutoTors program in AutoDock 4.0 and allowed to rotate (full ligand flexibility). For each docking, the initial position and conformation of the ligand were assigned randomly and the following docking parameters, similar to those validated previously for TbREL1 [12], were used: population size of 200, 7 x 10^6^ evaluations, 2.7 x 10^4^ generations, elitism of 1, rate of gene mutation 0.02, rate of crossover 0.8, and a local search rate of 0.06. The contribution for the intramolecular energy of the unbound ligand was performed for the extended conformation. Clustering of the predicted poses was performed with a cutoff of 2.0 Å root-mean-square deviation (RMSD).

**Figure Generation**

Figure 1 was generated in Adobe Illustrator CS3. Figures S1, S2, and S3 were generated by visualizing protein and ligand models in VMD 1.6.8, rendering the scene with Tachyon Ray Tracer 0.98, modifying color, contrast, and/or level balance in Adobe Photoshop CS3, and adding text labels in Adobe Illustrator CS3.

**References**

1. Coleman RG, Sharp KA (2006) Travel depth, a new shape descriptor for macromolecules: application to ligand binding. J Mol Biol 362: 441-458.

2. Nayal M, Honig B (2006) On the nature of cavities on protein surfaces: application to the identification of drug-binding sites. Proteins 63: 892-906.

3. Coleman RG, Burr MA, Souvaine DL, Cheng AC (2005) An intuitive approach to measuring protein surface curvature. Proteins 61: 1068-1074.

4. Agarwal PK, Edelsbrunner H, Harer J, Wang Y (2004) Extreme elevation on a 2-manifold. . Symp Comp Geo 20: 357-365.

5. Hendrix DK, Kuntz ID (1998) Surface solid angle-based site points for molecular docking. Pac Symp Biocomput: 317-326.

6. Liang J, Edelsbrunner H, Woodward C (1998) Anatomy of protein pockets and cavities: measurement of binding site geometry and implications for ligand design. Protein Sci 7: 1884-1897.

7. Norel R, Wolfson HJ, Nussinov R (1999) Small molecule recognition: solid angles surface representation and molecular shape complementarity. Comb Chem High Throughput Screen 2: 223-237.

8. Watson JD, Laskowski RA, Thornton JM (2005) Predicting protein function from sequence and structural data. Curr Opin Struct Biol 15: 275-284.

9. Xie L, Bourne P (2007) A robust and efficient algorithm for the shape description of protein structures and its application in predicting ligand binding sites. BMC Bioinformatics 8: S9.

10. Morris GM, Goodsell DS, Halliday RS, Huey R, Hart WE, et al. (1998) Automated docking using a Lamarckian genetic algorithm and an empirical binding free energy function. Journal of Computational Chemistry 19: 1639-1662.

11. Sousa SF, Fernandes PA, Ramos MJ (2006) Protein-ligand docking: current status and future challenges. Proteins 65: 15-26.

12. Amaro RE, Schnaufer A, Interthal H, Hol W, Stuart KD, et al. (2008) Discovery of drug-like inhibitors of an essential RNA-editing ligase in Trypanosoma brucei. Proceedings of the National Academy of Sciences 105: 17278-17283.

13. Oostenbrink BC, Pitera JW, van Lipzig MM, Meerman JH, van Gunsteren WF (2000) Simulations of the estrogen receptor ligand-binding domain: affinity of natural ligands and xenoestrogens. J Med Chem 43: 4594-4605.

14. Oostenbrink C, van Gunsteren WF (2004) Free energies of binding of polychlorinated biphenyls to the estrogen receptor from a single simulation. Proteins 54: 237-246.

15. Kim JT, Hamilton AD, Bailey CM, Domaoal RA, Wang L, et al. (2006) FEP-guided selection of bicyclic heterocycles in lead optimization for non-nucleoside inhibitors of HIV-1 reverse transcriptase. J Am Chem Soc 128: 15372-15373.

16. Bursulaya BD, Totrov M, Abagyan R, Brooks CL, 3rd (2003) Comparative study of several algorithms for flexible ligand docking. J Comput Aided Mol Des 17: 755-763.

17. Ewing TJ, Makino S, Skillman AG, Kuntz ID (2001) DOCK 4.0: search strategies for automated molecular docking of flexible molecule databases. Journal of computer-aided molecular design 15: 411.

18. Rarey M, Kramer B, Lengauer T, Klebe G (1996) A fast flexible docking method using an incremental construction algorithm. Journal of Molecular Biology 261: 470.

19. Jones G, Willett P, Glen RC, Leach AR, Taylor R (1997) Development and validation of a genetic algorithm for flexible docking. Journal of Molecular Biology 267: 727.

20. Roper JR, Guther ML, Milne KG, Ferguson MA (2002) Galactose metabolism is essential for the African sleeping sickness parasite Trypanosoma brucei. Proceedings of the National Academy of Sciences of the United States of America 99: 5884-5889

21. Urbaniak MD, Tabudravu JN, Msaki A, Matera KM, Brenk R, et al. (2006) Identification of novel inhibitors of UDP-Glc 4'-epimerase, a validated drug target for african sleeping sickness. Bioorg Med Chem Lett 16: 5744-5747.

22. Tomiya N, Ailor E, Lawrence SM, Betenbaugh MJ, Lee YC (2001) Determination of nucleotides and sugar nucleotides involved in protein glycosylation by high-performance anion-exchange chromatography: sugar nucleotide contents in cultured insect cells and mammalian cells. Anal Biochem 293: 129-137.

23. Attali C, Frolet C, Durmort C, Offant J, Vernet T, et al. (2008) Streptococcus pneumoniae choline-binding protein E interaction with plasminogen/plasmin stimulates migration across the extracellular matrix. Infect Immun 76: 466-476.

24. Miinalainen IJ, Chen ZJ, Torkko JM, Pirila PL, Sormunen RT, et al. (2003) Characterization of 2-enoyl thioester reductase from mammals. An ortholog of YBR026p/MRF1'p of the yeast mitochondrial fatty acid synthesis type II. J Biol Chem 278: 20154-20161.

25. Chen ZJ, Pudas R, Sharma S, Smart OS, Juffer AH, et al. (2008) Structural enzymological studies of 2-enoyl thioester reductase of the human mitochondrial FAS II pathway: new insights into its substrate recognition properties. J Mol Biol 379: 830-844.

26. Larkin MA, Blackshields G, Brown NP, Chenna R, McGettigan PA, et al. (2007) Clustal W and Clustal X version 2.0. Bioinformatics 23: 2947-2948.

27. Berman HM, Westbrook J, Feng Z, Gilliland G, Bhat TN, et al. (2000) The Protein Data Bank. Nucleic Acids Research 28: 235-242.

28. Bairoch A, Apweiler R, Wu CH, Barker WC, Boeckmann B, et al. (2005) The Universal Protein Resource (UniProt). Nucleic Acids Research 33: D154-159.

29. Ye Y, Godzik A (2003) Flexible structure alignment by chaining aligned fragment pairs allowing twists. Bioinformatics (Oxford, England) 19 Suppl 2: ii246.

30. Roberts E, Eargle J, Wright D, Luthey-Schulten Z (2006) MultiSeq: unifying sequence and structure data for evolutionary analysis. BMC bioinformatics 7: 382.

31. Humphrey W, Dalke A, Schulten K (1996) VMD: visual molecular dynamics. J Mol Graph 14: 33-38, 27-38.

| Confirmed Docking to an Active Site of Known Pharmacological Activity, and Homologs | | | | |
| --- | --- | --- | --- | --- |
|  | Receptor | Binding Energy | Name | Species |
| 1 | 2IBN:B | -30.18 | inositol oxygenase | H. sapiens |
| 2 | 1WRA:A | -28.00 | teichoic acid phosphorylcholine esterase/choline | S. pneumoniae |
| 3 | 2HD1:B | -18.19 | phosphodiesterase 9A | H. sapiens |
| 4 | 1I3L:B | -11.22 | UDP-galactose 4-epimerase | H. sapiens |
|  | 1GY8:D | -10.29 | UDP-galactose 4-epimerase | T. brucei |
|  | 2C20:E | -9.82 | UDP-glucose 4-epimerase | B. anthracis |
| 5 | 1SJ9:E | -11.19 | uridine phosphorylase | S. typhimurium |
| 6 | 2CH5:C | -10.75 | NAGK | H. sapiens |
| 7 | 2FAO:A | -10.57 | probable ATP-dependent DNA ligase | P. aeruginosa |
|  | 1X9N:A | -9.70 | DNA ligase I | H. sapiens |
|  | 1TAE:B | -9.49 | DNA ligase, NAD-dependent | E. faecalis v583 |
|  | 1ZAU:A | -6.75 | DNA ligase | M. tuberculosis |
|  | 1VS0:B | -9.03 | putative DNA ligase-like RV0938/MT0965 | M. tuberculosis |
| 8 | 2GR9:B | -10.49 | pyrroline-5-carboxylate reductase 1 | H. sapiens |
| 9 | 2HV7:C | -10.43 | phosphatase 2B, regulatory subunit B | H. sapiens |
|  | 2G62:A | -8.48 | phosphatase 2B, regulatory subunit B' | H. sapiens |
|  | 2IXM:A | -7.95 | serine/threonine-phosphatase 2a | H. sapiens |
| 10 | 2HJW:A | -10.28 | acetyl-CoA carboxylase 2 | H. sapiens |
| 11 | 2FIM:B | -10.21 | tubby related 1 | H. sapiens |
|  | 1S31:A | -9.17 | tubby isoform A | H. sapiens |
| 12 | 1XK5:A | -10.10 | snurportin-1 | H. sapiens |
| 13 | 1ZSY:A | -10.04 | mitochondrial 2-enoyl thioester reductase | H. sapiens |
| 14 | 2O8R:A | -10.01 | polyphosphate kinase | P. gingivalis |
| 15 | 1CI7:B | -9.85 | thymidylate synthase | P. carinii |
|  | 1HVY:D | -9.81 | thymidylate synthase | H. sapiens |
|  | 2AAZ:O | -9.71 | thymidylate synthase | C. neoformans |
| 16 | 1VHJ:B | -9.67 | purine nucleoside phosphorylase | V. cholerae |
|  | 1Z37:A | -9.41 | purine nucleoside phosphorylase | T. vaginalis |
|  | 1XE3:B | -9.21 | purine nucleoside phosphorylase | B. anthracis |
|  | 2AC7:A | -8.48 | purine nucleoside phosphorylase | B. cereus g9241 |
|  | 2B94:A | -7.61 | purine nucleoside phosphorylase | P. knowlesi |
|  | 2BSX:A | -7.54 | purine nucleoside phosphorylase | P. falciparum |
| 17 | 1P77:A | -9.64 | shikimate 5-dehydrogenase | H. influenzae |
| 18 | 1KET:B | -9.55 | dTDP-D-glucose 4,6-dehydratase | S. suis |
|  | 1KEU:B | -10.95 | dTDP -D-glucose 4,6-dehydratase | S. enterica subsp. enterica |
|  | 1G1A:C | -9.24 | dTDP -D-glucose 4,6-dehydratase | S. enterica |
| 19 | 1FZE:B | -9.53 | fibrinogen | H. sapiens |
| 20 | 1J3I:C | -9.53 | bifunctional dihydrofolate reductase-thymidylate | P. falciparum |
| 21 | 1F8W:A | -9.41 | NADH peroxidase | E. faecalis |
| 22 | 1TED:D | -9.13 | PKS18 | M. tuberculosis |
| 23 | 1EWN:A | -9.09 | 3-methyl-adenine DNA glycosylase | H. sapiens |
| 24 | 2A9G:A | -8.99 | arginine deiminase | P. aeruginosa |
| 25 | 2C8J:B | -8.87 | ferrochelatase 1 | B. anthracis |
| 26 | 2PH5:A | -8.62 | homospermidine synthase | L. pneumophila subsp. |
| 27 | 1MZV:A | -8.61 | adenine phosphoribosyltransferase | L. tarentolae |
|  | 1QCD:A | -9.90 | adenine phosphoribosyltransferase | L. donovani |
| 28 | 2HO3:C | -8.60 | oxidoreductase, GFO/IDH/MOCA family | S. pneumoniae |
| 29 | 2BCP:B | -8.57 | NADH oxidase | S. pyogenes |
| 30 | 1UKC:A | -8.56 | ESTA | A. niger |
| 31 | 2OEG:A | -8.56 | UTP-glucose-1-phosphate uridylyltransferase 2, | L. major |
| 32 | 1P16:A | -8.38 | mRNA capping enzyme alpha subunit | C. albicans |
| 33 | 1QTR:A | -8.31 | prolyl aminopeptidase | S. marcescens |
| 34 | 1WM1:A | -7.01 | proline iminopeptidase | S. marcescens |
| 35 | 2NR9:A | -6.44 | GLPG homolog | H. influenzae |
|  |  |  |  |  |
| Docked to Active Site of Known Pharmacological Activity Not Confirmed, and Homologs | | | | |
|  | Receptor | Binding Energy | Name | Species |
| 36 | 1K8T:A | -12.16 | calmodulin-sensitive adenylate cyclase | B. anthracis |
| 37 | 1CVR:A | -12.1 | gingipain R | P. gingivalis |
| 38 | 1YVH:A | -11.79 | CBL e3 ubiquitin ligase | H. sapiens |
| 39 | 2A74:D | -11.43 | complement component C3C | H. sapiens |
| 40 | 1X1F:A | -11.16 | signal-transducing adaptor 1 | H. sapiens |
| 41 | 2I07:A | -10.58 | complement C3B | H. sapiens |
| 42 | 1D5R:A | -10.57 | phosphoinositide phosphotase PTEN | H. sapiens |
| 43 | 2CBL:A | -10.48 | CBL | H. sapiens |
| 44 | 2OR4:A | -10.28 | glutamate carboxypeptidase 2 | H. sapiens |
| 45 | 1JVW:A | -10.17 | macrophage infectivity potentiator | T. cruzi |
| 46 | 1IJQ:B | -9.95 | low-density liporeceptor | H. sapiens |
| 47 | 1T29:A | -9.80 | breast cancer type 1 susceptibility | H. sapiens |
| 48 | 1FZC:E | -9.67 | fibrin | H. sapiens |
| 49 | 3BTA:A | -9.65 | botulinum neurotoxin type A | C. botulinum |
| 50 | 2B81:A | -9.60 | luciferase-like monooxygenase | B. cereus |
| 51 | 1URJ:B | -9.58 | major DNA-binding | H. herpesvirus 1 |
| 52 | 1PEO:A | -9.46 | ribonucleoside-diphosphate reductase 2 alpha | S. typhimurium |
| 53 | 2OIT:A | -9.35 | nucleoporin 214KDA | H. sapiens |
| 54 | 2HQQ:A | -9.31 | ketohexokinase | H. sapiens |
| 55 | 2HYE:A | -9.26 | DNA damage-binding 1 | H. sapiens |
| 56 | 1IMV:A | -9.23 | pigment epithelium-derived factor | H. sapiens |
| 57 | 2JOG:A | -9.18 | calmodulin-dependent calcineurin a subunit alpha | H. sapiens |
| 58 | 1S95:B | -9.05 | serine / threonine phosphatase 5 | H. sapiens |
| 59 | 2B3Y:B | -9.05 | iron-responsive element binding 1 | H. sapiens |
| 60 | 2POD:B | -8.95 | mandelate racemase / muconate lactonizing enzyme | B. pseudomallei |
| 61 | 1D3G:A | -8.93 | dihydroorotate dehydrogenase | H. sapiens |
|  | 2FPV:A | -8.94 | dihydroorotate dehydrogenase, mitochondrial | H. sapiens |
| 62 | 1L1O:A | -8.82 | replication A 14 KDA subunit | H. sapiens |
| 63 | 1T77:D | -8.69 | lipopolysaccharide-responsive and beige-like | H. sapiens |
| 64 | 2I4T:A | -8.65 | tricomonas vaginalis purine nucleoside | T. vaginalis |
| 65 | 2POF:B | -8.61 | CDP-diacylglycerol pyrophosphatase | E. coli o157:h7 |
| 66 | 2DE0:X | -8.53 | alpha-1,6-fucosyltransferase | H. sapiens |
| 67 | 1CPM:A | -8.44 | circularly permuted | P. macerans |
| 68 | 1MI1:B | -8.43 | neurobeachin | H. sapiens |
| 69 | 1R9J:A | -8.35 | transketolase | L. mexicana mexicana |
| 70 | 1T2B:B | -8.35 | P450CIN | C. braakii |
| 71 | 2BHO:A | -8.13 | SYCT | Y. enterocolitica |
| 72 | 1QUQ:B | -8.11 | replication A 32 KD subunit | H. sapiens |
| 73 | 1Q8M:D | -8.09 | triggering receptor expressed on myeloid cells 1 | H. sapiens |
| 74 | 2OLS:A | -8.08 | phosphoenolpyruvate synthase | N. meningitidis serogroup b |
| 75 | 1RW2:A | -8.00 | ATP-dependent DNA helicase II, 80 KDA subunit | H. sapiens |
| 76 | 2OBV:A | -7.95 | S-adenosylmethionine synthetase isoform type-1 | H. sapiens |
| 77 | 2IBI:A | -7.93 | ubiquitin carboxyl-terminal hydrolase 2 | H. sapiens |
| 78 | 1W9E:A | -7.82 | syntenin 1 | H. sapiens |
| 79 | 1N1F:A | -7.49 | interleukin-19 | H. sapiens |
| 80 | 1S0F:A | -7.28 | botulinum neurotoxin type B | C. botulinum |
| 81 | 1CZ1:A | -7.15 | EXO-B-1,3-glucanase | C. albicans |
| 82 | 1Q5N:A | -7.08 | 3-carboxy-cis,cis-muconate cycloisomerase | A. calcoaceticus |
| 83 | 2B5L:A | -6.79 | damage-specific DNA binding 1 | H. sapiens |
| 84 | 2I0M:A | -6.69 | phosphate transport system PHOU | S. pneumoniae |
| 85 | 1KEH:A | -6.30 | precursor of cephalosporin acylase | B. diminuta |
| 86 | 1QGU:B | -6.10 | nitrogenase molybdenum iron | K. pneumoniae |
| 87 | 1ZZ1:B | -5.91 | histone deacetylase-like amidohydrolase | A. bacterium |

**Table S1.** Predicted secondary targets of **1**.
