# Supplementary figures and images for: A Multidimensional Strategy to Detect Polypharmacological Targets in the Absence of Structural and Sequence Homology
Source: PLoS Comput Biol. 2010 Jan 22;6(1):e1000648. doi: 10.1371/journal.pcbi.1000648 (PMC2799658; doi:10.1371/journal.pcbi.1000648)

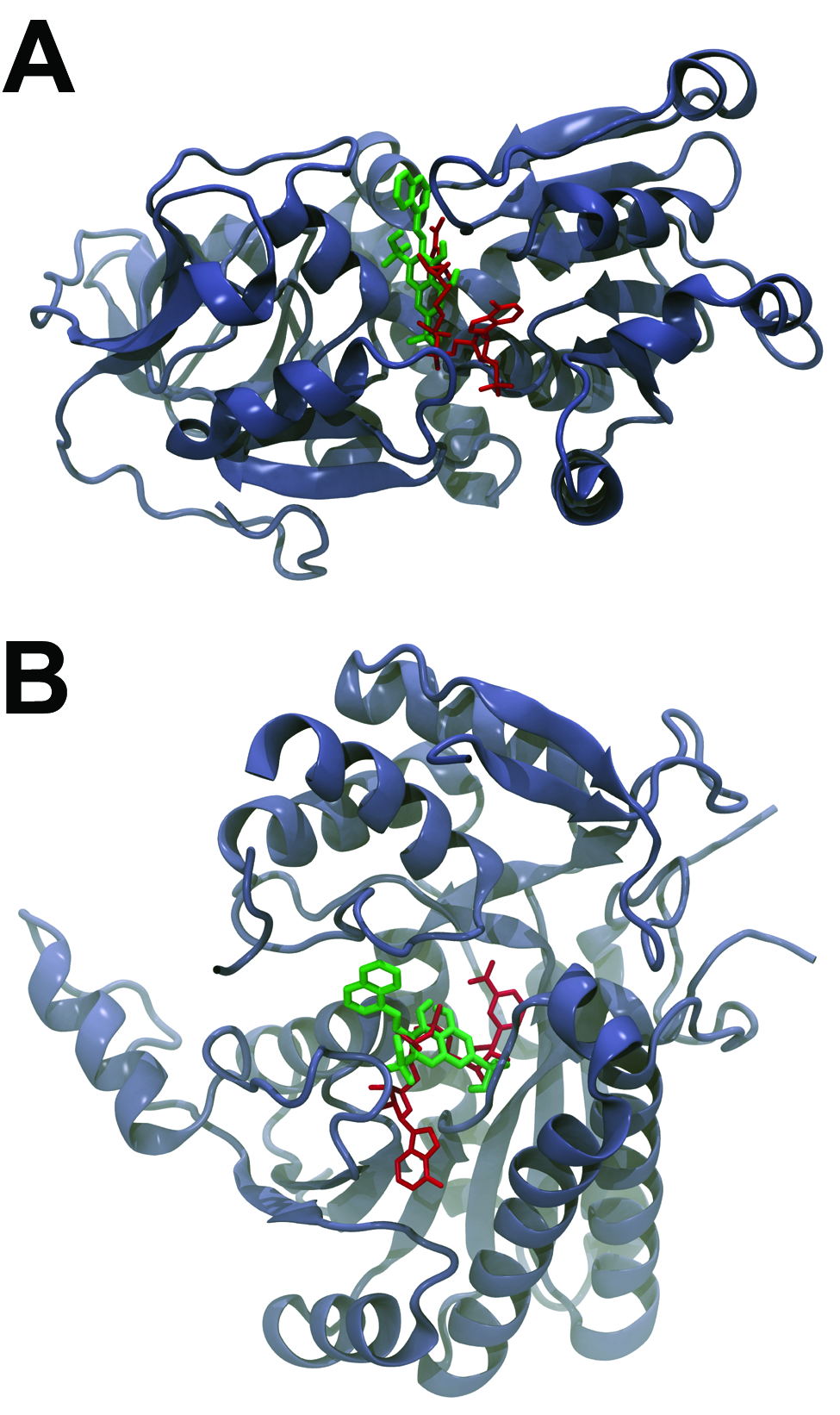

Supplement: Figure S1 — Possible binding of compound 1 in NAD+ and NADPH pockets. In the case of two experimentally validated secondary targets, AutoDock predicted that 1 would bind in a NAD+ or NADPH pocket, suggesting that 1 may be a competitive inhibitor for these co-factors. (a) HsETR1. The crystal structure (PDB: 1ZSY) contained no NAD+ co-factor, so a related structure (PDB: 1GUF) with co-crystallized NDP (NADPH dihydro-nicotinamide-adenine-dinucleotide phosphate) was aligned to the 1ZSY structure using MultiSeq. The aligned NDP is shown in red. The predicted binding pose of 1 is shown in green. (b) TbGalE. The co-crystallized NAD+ co-factor is shown in red. The predicted binding pose of 1 is shown in green. (3.98 MB TIF) [file pcbi.1000648.s002.tif]

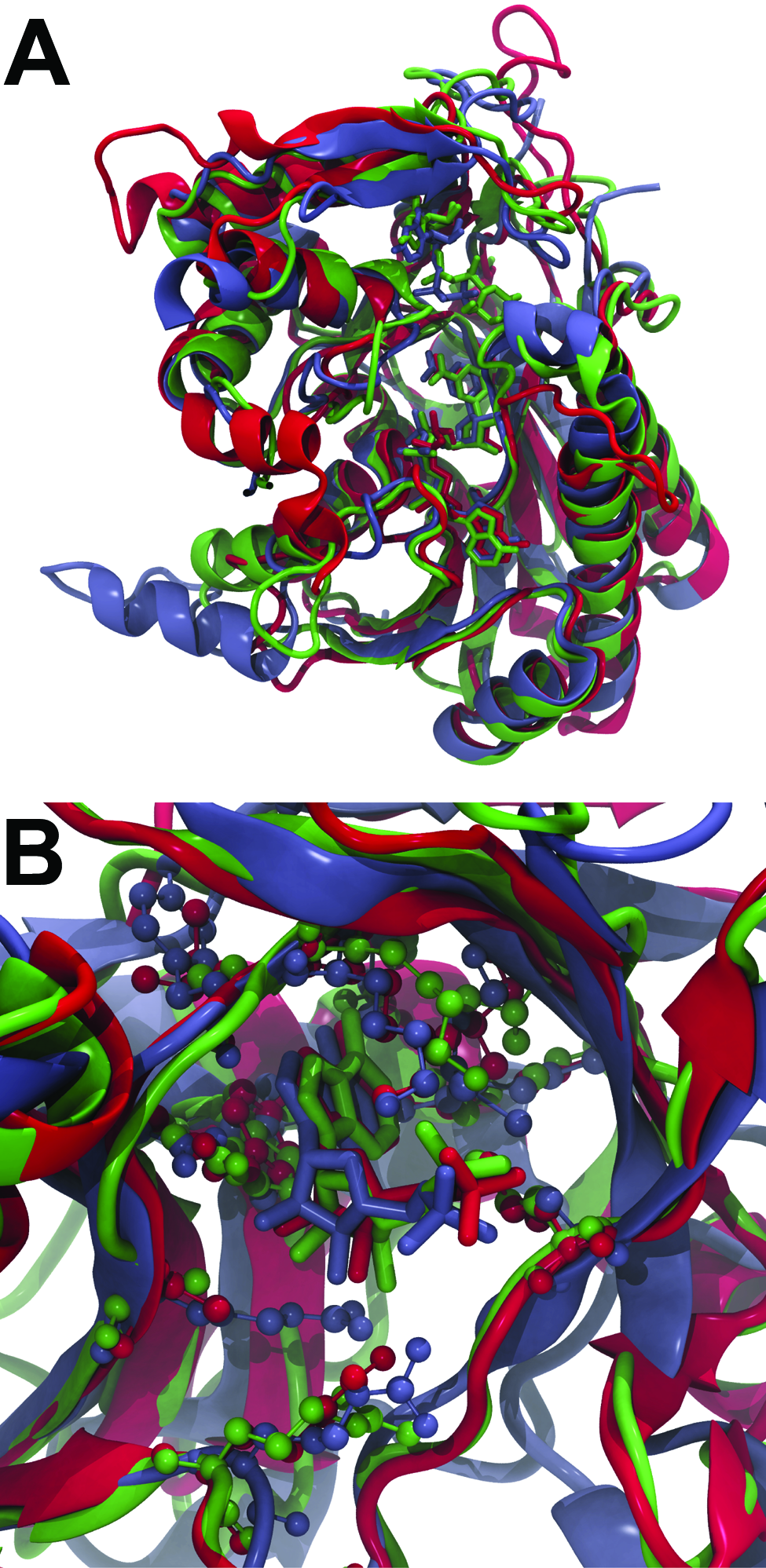

Supplement: Figure S2 — Unvalidated but likely secondary targets of compound 1. (a) The current strategy correctly identified TbGalE as a secondary target of 1. Additionally, HsGalE and SeRmlB, both TbGalE homologs, are also predicted to be off-target receptors. SeRmlB and TbGalE were aligned to HsGalE using MultiSeq to demonstrate structural similarity. Blue: TbGalE; Red: SeRmlB; Green: HsGalE. (b) The current strategy identified a number of DNA ligases as predicted secondary targets of 1. Three of these DNA ligases are homologous with HsLigIIIβ, an experimentally validated secondary target. The structures of the three predicted secondary targets were aligned using MultiSeq to demonstrate structural similarity. Portions of some ligands were removed to simplify visualization. The active site is shown with selected protein residues to demonstrate active-site similarity. Blue: HsLigI; Red: Ef ligase; Green: Mt ligase. (3.65 MB TIF) [file pcbi.1000648.s003.tif]

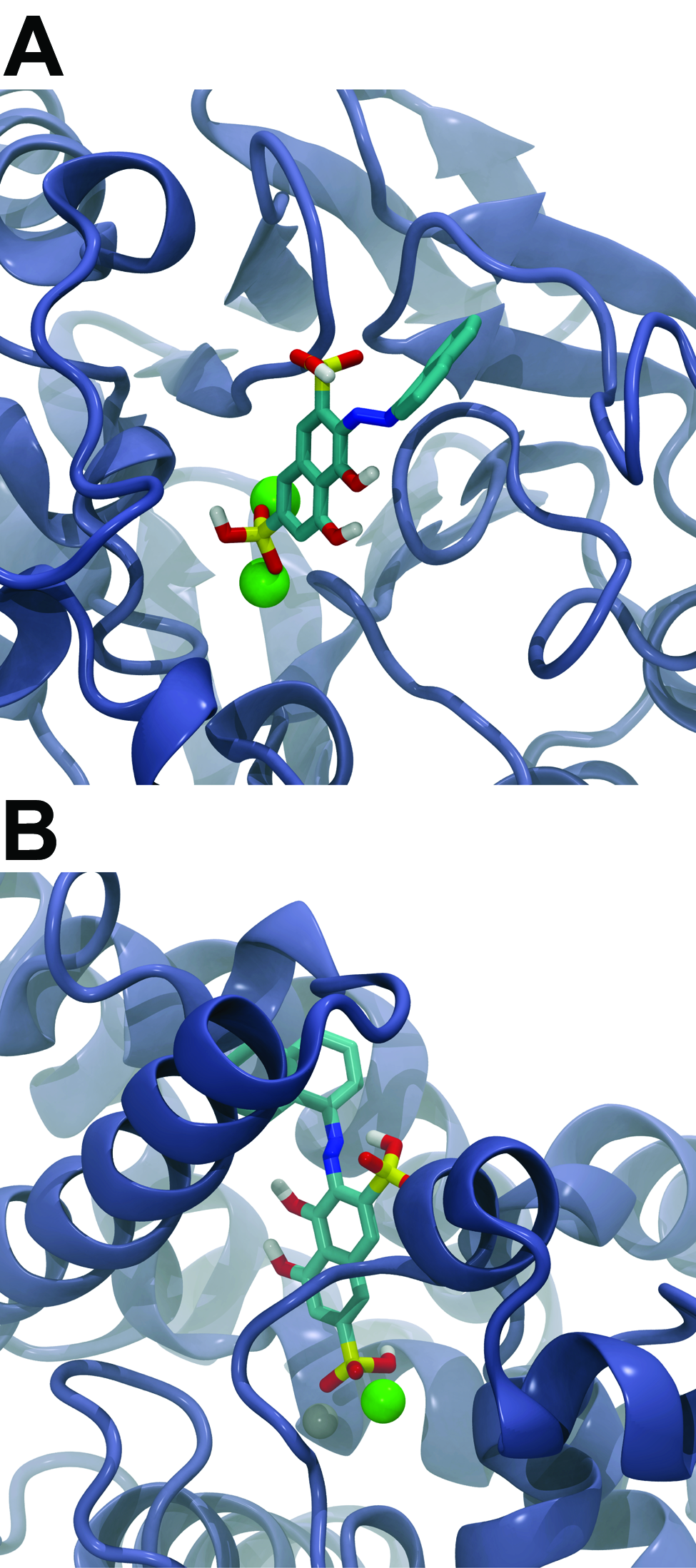

Supplement: Figure S3 — False-positive predictions. Compound 1 is shown in licorice, docked into each protein crystal structure. Magnesium and zinc are shown in green and grey, respectively. In both cases, one of the sulfonate groups of 1 is juxtaposed against multiple metal cations, leading to an exaggerated estimate of the electrostatic energy. (a) SpPce was predicted to bind 1 with −28.00 kcal/mol. (b) HsPDE9A2 was predicted to bind 1 with −18.19 kcal/mol. (2.66 MB TIF) [file pcbi.1000648.s004.tif]
